# The effect of unhealthy dietary habits on the incidence of dental caries and overweight/obesity among Egyptian school children (A cross-sectional study)

**DOI:** 10.3389/fpubh.2022.953545

**Published:** 2022-08-16

**Authors:** Sara Ahmed Mahmoud, Sara El Moshy, Dina Rady, Israa Ahmed Radwan, Marwa M. S. Abbass, Ayoub Al Jawaldeh

**Affiliations:** ^1^Pediatric Dentistry and Dental Public Health Department, Faculty of Dentistry, Cairo University, Cairo, Egypt; ^2^Oral Biology Department, Faculty of Dentistry, Cairo University, Cairo, Egypt; ^3^World Health Organization (WHO), Regional Office for the Eastern Mediterranean (EMRO), Cairo, Egypt

**Keywords:** unhealthy diet, dental caries, school children, overweight, obesity

## Abstract

**Background:**

Obesity and dental caries are public health problems in Egypt. Factors such as unhealthy diet, poor oral hygiene, and physical inactivity can play a major role in both problems. This study was carried out to illuminate the mutual unhealthy dietary risk factors associated with the incidence of both health conditions.

**Methods:**

Between 1 October 2020 and 1 July 2021, 369 Egyptian children (5–10 years) were examined. Dental status was assessed using decayed, missing/extracted, and filled tooth indices (dmft, deft, and DMFT) for deciduous, mixed, and permanent dentitions, respectively. Moreover, the lifestyle, food habits, and body mass index (BMI) were recorded.

**Results:**

A total of 342 (93.7%) of the included subjects suffered from caries, and only 27(7.3%) were caries-free. Based on BMI percentiles, 247 (66.9%) of the youngsters were overweight/obese, while 122 (33.1%) had normal weight. The mean dmft was 6.9 (±4.6), deft 4.2 (±3.3), and DMFT 0.1 (±1.7). In the primary dentition, a significant positive correlation was detected between dmft and BMI, legumes, sweetened milk and juice, soft drinks, and desserts, while a significant negative correlation was detected between dmft/deft, meat/poultry/fish, fresh fruits, and vegetables. A significant positive correlation was detected between deft and BMI, sweetened milk and juice, ice cream, candies, and crackers. In the permanent dentition, a significant positive correlation was detected between age, soft drinks, sweetened juice, desserts, and DMFT, while a significant negative correlation was detected with fresh fruits and vegetables. BMI was significantly negatively correlated with a healthy lifestyle, meat/poultry/fish consumption, and fresh fruits and vegetables while positively correlated with legumes, ice cream, soft drinks, granulated sugars, desserts, fast food, and caffeinated drinks.

**Conclusion:**

Overweight/obesity was positively correlated with primary dentition dental caries. Desserts (sweetened snacks) and soft drinks could be the common risk factors associated with high caries and overweight/obesity incidence among Egyptian school children; conversely, consumption of fruits and vegetables could hinder both health conditions. Moreover, sweetened juices were associated with primary and permanent dental caries.

## Introduction

Dental caries and obesity are considered worldwide growing public health problems that significantly impact children's lives and impose an enormous cost on society (1, 2). The Global Burden of Disease Study 2017 revealed that oral diseases affect 3.5 billion people around the world (3). Dental caries is considered the most common chronic disease affecting 2.43 billion people globally (4). Caries in deciduous teeth affects more than 530 million children worldwide (3). Despite the global spread of the disease, its incidence exhibits geographical diversity across developing and developed countries. It has been asserted that dental caries is decreasing in most industrialized nations due to enhanced prevention programs and expanded access to dental health services. Even so, contradictory results suggest that dental caries is still prevalent among underprivileged communities in many of these nations (5–7). In most developing countries, dental caries levels were low until recent years. An increase was observed due to rising sugar consumption, inadequate fluoride exposure, and limited access to oral healthcare services (5, 6, 8). The Egyptian Ministry of Health and the World Health Organization (WHO) in 2014 conducted an oral health survey in Egypt and revealed that approximately 70% of children had untreated caries (9). In 2022, the comparison between different governorates showed that the highest dental caries prevalence was in children living in Cairo (85%), while for those living in upper Egypt and Deltas was lower (82% and 83.5, respectively) (10).

Children with dental caries have significantly impaired social and psychological functioning. Pain in the teeth, mouth, or jaws, irritation or frustration, difficulty in eating, and sleeping are the most common effects reported by parents in the literature (11–15). Poor dental hygiene greatly impacts the child's growth and cognitive development over time by interfering with the child's nutrition which results in reduced body mass and stature (16–20). Subsequently, the impacts include school absences, inability to concentrate in school, decreased self-esteem, poor social relations, impaired speech development, sleep problems, and inadequate nutrition (21). Family members also suffer when a child has untreated dental caries, including the caretaker's inability to get enough rest, the caretaker missing time at work, and the caretaker's stress and financial hardship due to the time and money required to get the child to the dentist (11, 12, 14, 15).

The WHO in 2003 established global goals for oral health to guide health planners and policymakers in improving the oral health status of their populations (22). Subsequently, the WHO published its global action plan for preventing and controlling non-communicable diseases (NCDs) 2013–2020 (23). The action plan has two goals; the first is to start reducing risk factors for NCDs and underlying social determining factors through developing health-promoting environments; second, to strengthen and direct health systems to prevent and control NCDs and the social factors that cause them through people-centered primary healthcare and universal health coverage. The WHO resolution on Oral Health in 2021 reinforces these goals regarding oral diseases and dental caries. This occurs by encouraging countries to abandon the traditional curative approach in favor of a “preventive promotional approach with risk identification for timely, comprehensive, and inclusive care, taking into account all stakeholders in contributing to the improvement of the population's oral health with a positive impact on overall health” (24, 25).

Obesity is a major public health concern affecting over 650 million people globally (26). According to WHO, Egypt ranks 18th globally in the prevalence of obesity (27). The prevalence of overweight among children and adolescents aged 5–19 has significantly risen in Egypt from 22.6% in 2000 to 36.7% in 2016. Among EMR countries that suffer from a high incidence of overweight among children, Egypt has been ranked third after Kuwait and Qatar. In addition, the prevalence of obesity among children and adolescents aged 5–19 in Egypt has nearly doubled between 2000 and 2016 from 9 to 17.6% (28). The WHO in 2016 reported that more than 340 million children and adolescents between the ages of 5 and 19 are classified overweight or obese (26). Moreover, a report by WHO in 2018 revealed that the worldwide prevalence of obesity in children and adolescents had increased more than 10-fold in the last four decades (29). Despite the WHO efforts, the expanded utilization of unhealthy diets led to an increased prevalence of dental caries and childhood overweight/obesity worldwide in the past decades (30–32).

Dietary sugar specifically becomes a significant public health issue with concerns regarding its contribution to increased obesity prevalence and its negative impact on oral health (33, 34). Sweetened foods and soft drinks are the first choice of children as snacks between meals (35). These food items are rich in carbohydrates and thus result in an increased risk for caries development (36).

Both dental caries and obesity possess common characteristics of being chronic and highly prevalent conditions (5, 37, 38). They are also thought to have the same contributing factors, including genetic, biological, dietary, socioeconomic, cultural, and lifestyle predisposing factors (39, 40). Diet plays an essential role in the development of dental caries and obesity where a high consumption of sugar-sweetened beverages, junk foods, and fermentable carbohydrates influences their incidence (41, 42). Moreover, lifestyle may contribute to the development of obesity and dental caries also by increasing the time spent on social media and watching TV while consuming unhealthy snacks, which can lead to a reduction in physical activity time (43, 44).

Furthermore, both conditions are more prevalent in specific communities with low socioeconomic status in association with the low education level of the parents, unhealthy diet consumption, and the difficulty in acquiring adequate healthcare and services (41, 45, 46). The results of our previous study emphasize this assumption as SES, parental educational, and oral hygiene measures were significantly inversely correlated with primary teeth dental caries, whereas permanent teeth caries revealed non-significant correlations (47). Conversely, low and high socioeconomic status were demonstrated to increase the risk of dental caries, while a middle socioeconomic status had a 20% lesser chance in low- and middle-income countries. It was reported that 44% of the children had access to dental care services, which implies the role of numerous socioeconomic constraints in addition to geographical restrictions (48). Moreover, the relation between obesity and socioeconomic status depends on the stage of the nutrition transition (49). In nutrition, the concept of “transitions” has been used to describe trends in significant population health parameters to provide insight into underlying determinants, positive deviations, and future directions (50). Analysis of Egypt's Demographic and Health Survey data between 1992-1995 and 2005-2008 revealed that the greatest relative increases in the prevalence of obesity occurred among women with no/primary education and in the lowest income quintile (25).

The correlation between dental caries and obesity is a controversial issue. In literature, being overweight has been linked to an increased incidence of dental caries (51) due to the upregulated intake frequency of sugary foods and snacks (52). This correlation was supported by a systematic review that reported a significant relationship between childhood obesity and dental caries (53). On the contrary, being underweight has also been associated with high caries prevalence (54, 55), as the pain resulting from dental caries could make the child eat less food (17, 56). Other studies reported that dental caries was not correlated with obesity (57, 58).

Estimating the burden of the unhealthy diet that could contribute to the incidence of dental caries and obesity is crucial in determining the public health intervention priorities. It also helps educate the public about the negative effects of dental caries and obesity and provides health policy decision makers with information about the scope of health problems. The impact of unhealthy diets in correlation to dental caries and obesity among the Egyptian population is not well–established. Therefore, this study aims to evaluate the impact of unhealthy dietary habits on the incidence of dental caries and overweight/obesity among children aged 5 to 10 years to guide parents to be more conscious of their role in preventing these related health problems. Moreover, the need for high-quality and comparable data is a key component for the healthcare ministry to take essential actions regarding major health problems.

## Subjects and methods

The research was conducted following the Research Ethics Committee of Cairo University's Faculty of Dentistry's requirements (Approval: 24,920). The parents or guardians of the children gave their written informed consent to participate in the study. From 1 October 2020 to 1 July 2021, individuals were recruited from the Pedodontic outpatient clinics of Cairo University's Faculty of Dentistry. The criteria for the included children were as follows: age: ranges from 5 to 10 years old; Gender: males and females; Ethnicity: Egyptians. Children with visible disorders, physical or mental abnormalities, diabetes, or other systemic ailments or having orthodontic treatment were not included in the study. Children and parents who refused to participate were also excluded.

The sample size was calculated to be 369 using the basic formula (59). For dental caries, the prevalence in Egypt was estimated at 60% according to the WHO Regional Office for the Eastern Mediterranean (WHO EMRO) report in 2014 (9). Abbass et al. reported 74 % among children aged 3–18 years while reported 93.2 % among children aged 5–10 years (47). In EMR, caries was estimated by 66% (59–73%) for children aged 6–15 years (60). Moreover, 51.6% was reported among different Australian refugees (61). The average of the previously mentioned percentages was utilized in calculations; accordingly, the estimated sample size was 330. For overweight/obesity, Egypt's population of children aged (5–10) years was estimated at 30,000,000 (62), and the prevalence of overweight and obesity in this population was estimated at 40.2 % according to WHO data (63), and the estimated sample size was 369.

### Data collection and grouping

Name, age, gender, address, and type of education (government, experimental, or private) were among the sociodemographic data obtained from children's guardians. The children's lifestyle habits were documented, and the dietary habits were thoroughly reported using a food frequency questionnaire. The first two parts of the questionnaire that included the sociodemographic data and the dentition status were validated in WHO-oral health surveys (64), while the third part that included the lifestyle habits was validated in (65, 66). Finally, the part of the dietary habits was validated by Abbass et al. and the diet history questionnaire (47, 67–69). The assessed 18 dietary elements are included in Table 1.

**Table 1:** Description of investigated dietary elements.

**Dietary item**	**Full description**
Protein food	Meats/poultry and fish
Carbohydrates	Bread, rice, macaroni, mahshi, potatoes and sweet potatoes
Fruits/Vegetables	Fresh
Legumes	Favabeans, homos, wheat and peas
Sweetened cereals	Sweetened belyla, sweetened oats
Dairy products	Unsweetened milk, yogurt and cheese (all types) (butter is not included)
Sweetened milk	With or without flavors
Diary based ice cream	—
Soft drinks	pespsi, coke, fanta, 7up, sprite, etc…..
Fruits/Vegetables	Fresh
Sweetened juices	fresh/canned
Sugar	Sugar added to drinks (hot or cold), eaten sugar granules.
Candies	Hard, Sticky, lollipops
Jam, molasses and honey	Jam, molasses, honey and halawa
Crackers	Biscuits and chips
Desserts (sweetened snacks)	Cakes, doughnuts, sweetened pies, baklawa, basbosa, konafa
Junk food	Any of the following food items prepared outside home (ready to eat food): burgers, pizza, fried (potatoes, chicken, falafal), favabeans, koshary, shawarma sandwiches.
Chocolate	Bars, chocolate cakes
Soda	Sweetened carbonated drinks
Juices	Fresh, canned and citric,
Caffeinated drinks	Tea, coffee, Nescafe sachets (commonly known in Egypt as 3 in 1)

The authors filled out the questionnaire according to parents' answers on behalf of their children. The frequencies used in the questionnaire were once per month; 1–2 times per week; 3–4 times per week; 5–6 times per week; once per day; 2–3 times per day; 4–5 times per day; and 6 or more times per day. To facilitate the comparison and statistical analysis, these frequencies were merged into the three frequencies displayed: ≤ 2 times/week; 3–6 times/week; 1–6 times/day. According to WHO (1995) (70), body weights were measured using a Beurer scale (Ulm, Germany) with the participants wearing clothes but without shoes. Standing heights were measured to the nearest 0.1 cm utilizing a stadiometer. Body mass index (BMI) was calculated from the measured heights and weights. The obtained BMI values were plotted on the WHO percentile body mass index (BMI/age) charts for boys and girls (71). The children were divided into four categories based on their BMI percentiles: the underweight group (<5th percentile); the normal group (≥ 5^th^- <85^th^ percentile); the overweight group (≥ 85^th^- <95^th^ percentile); and the obese group (≥ 95th percentile). Moreover, children were divided into three groups based on their age: group I (5– <7 years old), group II (7– <9 years old), and group III (9, 10).

Furthermore, participants' responses to questions regarding lifestyle habits were marked, and a total score was calculated for each participant as previously described (72–75). Participants with a cumulative score of (0–3) were categorized into unhealthy lifestyle habits, while those with a cumulative score of (4–5) were categorized into moderate lifestyle habits, while participants with a cumulative score of (6–7) were categorized into healthy lifestyle habits.

### Oral examination

Examiners were trained and calibrated over 3 days in 3 sessions, with disparities in observations discussed among the examiners for reassessment and consensus (76, 77). Following the WHO guidelines, oral examination was performed on a dental chair in artificial light using a plain mouth mirror and a dental probe (78). During the clinical examination, all of the teeth that were present were considered (77).

Any lesion with a detectably softened floor, undermined enamel, or softened wall in a pit or fissure or on a smooth tooth surface, tooth surface containing a temporary filling requiring further treatment, and tooth surface containing a permanent restoration with an area of decay were all considered carious (either primary or secondary caries). The DMFT index, which measures the number of D (decayed tooth), M (missing tooth), and F (filled tooth), was used to determine the severity of caries in permanent teeth. The dmft index was employed for primary teeth: d (decayed teeth), m (missing teeth), and f (filled tooth). The deft index was employed for mixed dentition: d (decayed tooth indicated for filling), e (decayed tooth advised for extraction), and f (filled tooth) (64). The DMFT, dmft, and deft indices were divided into three categories: caries index zero (no carious teeth), caries index 1–3 (number of carious teeth 1–3), and caries index ≥4 (number of carious teeth ≥ 4) for statistical analysis (79).

### Statistical analysis

Data were statistically described as frequencies (number of cases) and percentages. Since data were non-parametric, the Kruskal–Wallis test was used for comparing more than two groups, while the Mann–Whitney test was used for comparing two groups. Spearman's rank correlation was used for the detection of correlation between different variables. Multivariate logistic regression analysis was used to test for the preferential effect of each food item on caries index and obesity after adjusting the effect of age and gender. *P*-values of < 0.05 were considered statistically significant. All statistical calculations were done using the computer program IBM SPSS (Statistical Package for the Social Science; IBM Corp, Armonk, NY, United States), release 22 for Microsoft Windows.

## Results

### Population profile

The study included 180 (48.8%) boys and 189 (51.2%) girls. About 172(46.6%) participants fell into the first age group AI (5–6.9 years), 97(26.3%) fell into the second age group AII (7–8.9 years), while 100 (27.1%) fell into the third age group AIII (9–10 years). The majority of participants 162 (43.9%) attended governmental schools. A total of 122 (33.1%) of the participants had normal weight, while 247 (67.0%) were overweight or obese. No underweight participants have been reported in this work; therefore, this category is not presented in the result section. About 342 (93.7%) of the included subjects suffered from dental caries, and only 27 (7.3%) individuals were caries-free (Table 2).

**Table 2:** Demographics, lifestyle habits and dietary habits.

**Parameter**	**Categories number (%)**				
**1- Age (years)**	**AI (5–6.9 years)**	**AII (7-8.9 years)**	**AIII (9-10 years)**
	172 (46.6%)	97 (26.3%)	100 (27.1%)
**2- Gender**	**Boys**	**Girls**	
	180 (48.8%)	189 (51.2%)	
**3-Children education**	**Governmental**	**Private**	**Experimental**	**Others**
	162 (43.9%)	100 (27.1%)	36 (9.8%)	71(19.2%)
**4- Body Mass Index**	**Normal (≥5- <85)**	**Overweight (≥85– <95) and Obese (≥95)**		
	122 (33.1%)	247 (66.9%)		
**5- Dental status**	**Caries free**	**Caries**		
	342 (93.7%)	27 (7.3%)		
**6- Lifestyle habits**	**Unhealthy habits**	**Moderate**	**Healthy habits**	
	152 (41.2%)	190 (51.5%)	27 (7.3%)	
a. **Breakfast meal**	**No**	**Sometimes**	**Most of the times**	**Rare**	**Unknown**
	34 (9.2%)	105 (28.5%)	207 (56.1%)	21(5.7%)	2(0.5%)
b. **Watching TV while eating**	**No**	**Sometimes**	**Most of the times**	**Rare**	**Unknown**
	62 (16.8%)	133 (36.0 %)	162 (43.9%)	9(2.4%)	3(0.8%)
c. **Eating outside home**	**No**	**Sometimes**	**Most of the times**	**Rare**	**Unknown**
	133 (36.0%)	159 (43.1%)	29 (7.9%)	46(12.5%)	2(0.5%)
d. **Eating before bedtime**	**Don't eat at night**	**One hour**	**2 hours**	**3 hours**	**Just before bed time**
	17 (4.6%)	131 (35.5%)	97 (26.3%)	52(14.1%)	72(19.5%)
e. **Family meals**	**Alone**	**All/some family**	**Other**	**Unknown**	
	36 (9.8%)	286 (77.5%)	30 (8.1%)	17 (4.6%)	
f. **Exercise /physical activity**	**No**	**Yes**	**Unknown**		
	227 (61.5%)	122 (33.1%)	20 (5.4%)		
g. **Using internet more than 2 hours per day**	**Yes**	**No**	**Unknown**		
	175 (47.4%)	178 (48.3%)	16 (4.3%)		
**7- Food consumption**	**≤2 times/week**	**3-6 times/week**	**1-6 times/day**		
a. **Meat/poultry/fish**	130 (35.2%)	127 (34.4%)	112 (30.4%)		
b. **Carbohydrates**	26 (7.1%)	73 (19.8%)	270 (73.2%)		
c. **Fresh fruits and vegetables**	96 (26.0%)	80 (21.7%)	193 (52.3%)		
d. **Legumes (fava beans/ hommos)**	164 (44.4%)	72 (19.5%)	133 (36.1%)		
e. **Sweetened cereals/ belela**	229 (62.1%)	44 (11.9%)	96 (26.0%)		
f. **Dairy products (unsweetened milk/ cheese/ yogurt**	85 (23.0%)	66 (17.9%)	218 (59.1%)		
g. **Sweetened milk**	183 (49.6%)	54 (14.68%)	132 (35.8%)		
h. **Dairy ice cream**	234 (63.5%)	71 (19.2%)	64 (17.3%)		
i. **Soft drinks**	243 (65.9%)	50 (13.6%)	76 (20.6%)		
j. **Sweetened juice**	175 (47.4%)	86 (23.3%)	108 (29.3%)		
k. **Granulated sugars**	270 (73.2%)	28 (7.6%)	71 (19.2%)		
l. **Candies (sticky or not)**	123 (33.3%)	67 (18.2%)	179 (48.5%)		
m. **Halawa/ honey**	202(54.7%)	60(16.3%)	107 (29.0%)		
n. **Crackers (biscuits/ chips)**	80 (21.7%)	62 (16.8%)	227 (61.5%)		
o. **Cakes/ doughnuts/ sweetened pies/ baklawa/ basbosa/ konafa**	250 (67.8%)	66 (17.9%)	53 (14.3%)		
p. **Fast food**	244 (66.1%)	67 (18.2%)	58 (15.7%)		
q. **Chocolate**	163 (44.1%)	70 (19.0 %)	136 (36.9%)		
r. **Caffeinated drinks (coffee, tea)**	268 (72.6%)	31 (8.4%)	70 (19.0 %)		

### Lifestyle habits

Most of the parents stated that their children had breakfast meal most of the time. In addition, 162 (43.9%) of the parents mentioned that their child would watch TV while eating, 159 (43.1%) of the parents stated that their children sometimes eat outside their home, and 131 (35.5%) of the participants would have their dinner 1 h before bedtime. Most of the parents 286 (77.5%) mentioned that the whole family had their meals together, and 227 (61.5%) acknowledged that their child does not perform any sort of physical exercise. A total of 175 (47.4%) of the participating children used the internet more than 2 h per day, while 178 (48.2%) did not. The majority of the participants 190 (51.5%) were categorized as moderate regarding lifestyle habits (Table 2).

### Dietary habits

Most of the participants consumed meat, legumes (fava beans/ hommos), sweetened cereals/ belela, sweetened milk, ice cream, soft drinks, sweetened juice, granulated sugars, desserts (cakes/ doughnuts/ sweetened pies/ baklawa/ basbosa/ konafa), fast food, chocolate, and caffeinated drinks (coffee, tea) less than or equal to 2 times/week, while the majority consumed carbohydrates, fresh fruits, and vegetables, dairy products (unsweetened milk/ cheese/ yogurt), candies, and crackers (biscuits/ chips), 1 to 6 times per day (Table 2).

### Mean decayed, missing/extracted, filled teeth (Dmft, deft, and DMFT)

The mean dmft recorded for the participants was 6.8 (±4.5), the mean deft was 4.2 (±3.3), while the mean DMFT was 0.9 (±1.7). The highest mean DMFT 1.8 (±2.0) was detected within AIII age group. In contrast, AI age group showed the highest mean dmft and deft of 6.9 (±4.5) and 5.5 (±3.9), respectively (Table 3).

**Table 3:** Mean (±SD) of dmft, deft, and DMFT indices.

**Variable**	**AGE**	**N**	**Mean**	**Variable**	**AGE**	**N**	**Mean**	**Variable**	**AGE**	**N**	**Mean**
**mean (±SD)**			**(±SD)**	**mean (±SD)**			**(±SD)**	**mean (±SD)**			**(±SD)**
dmft 6.829 (±4.541)	**AI**	105	6.9 (±4.6)	deft 4.2 (±3.3)	**AI**	67	5.5 (±3.9)	DMFT 0.1 (±1.7)	**AI**	67	0.09 (±0.4)
	**AII**	0			**AII**	97	4.7 (±3.1)		**AII**	97	0.8 (±1.4)
	**AIII**	0			**AIII**	100	2.8 (±2.5)		**AIII**	100	1.8 (±2.1)

### Correlation between caries indices and different risk factors

#### Correlation between dmft index and different risk factors

The majority of participants had dmft more than 4. The highest percentage of participants with dmft more than 4 were overweight or obese. The highest percentage of participants with dmft more than 4 were males. Most of the participants with dmft more than 4 had unhealthy life habits. No significant correlation was detected between dmft and gender or age, a statistically significant positive correlation was detected between dmft and BMI, while a statistically significant negative correlation was detected between dmft and healthy lifestyle habits (Table 4).

**Table 4:** Correlation between dmft index and different risk factors (Total number 105).

**Parameter and categories**		**Number (%)**			**M-W test**	**Correlation**		**Logistic regression**			
		**dmft = 0 7 (6.7%)**	**dmft = 1-3** **21 (20%)**	**dmft > 4 77 (73.3%)**	***p*-value**	**rho**	***p*-value**	***p*-value**	**OR**	**95%CI for OR**	
										**Lower**	**Upper**
**Age (years)**	**AI 105 (100%)**	7 (6.7%)	21 (20.2%)	77 (73.3%)	<0.05^*^	0.059	0.553				
	**AII 0**	0	0	0							
	**AIII 0**	0	0	0							
**Gender**	**Boys 56 (53.3%)**	2 (3.6%)	9 (16.1%)	45 (80.4%)	<0.05^*^	−0.177	0.071				
	**Girls 49 (46.6%)**	5 (10.2%)	12 (24.5%)	32 (65.3%)							
**BMI**	**Normal (≥5- <85) 34 (32.38%)**	7 (20.5%)	13 (38.2%)	14 (41.1%)	0.576	0.523	<0.05^*^				
	**Overweight (≥85- <95)**	0	8 (11.3%)	63 (88.7%)							
	**and Obese (≥95) 71 (67.61%) Lifestyle habits**
**Lifestyle habits**	**Unhealthy habits 44(41. 9%)**	0	7 (33.33%)	37 (48%)	<0.05^*^	−0.273	0.005^*^				
	**Moderate 50(47.6%)**	4 (57.1%)	11 (52.4%)	35 (45.5%)							
	**Healthy habits 11(10.5%)**	3 (42.8%)	3 (14.3%)	5 (6.5%)							

* Statistically significant.

The highest percentage of participants with dmft more than 4 consumed meat, sweetened cereals/ belela, sweetened milk, dairy ice cream, soft drinks, granulated sugar, halawa/ honey, desserts (cakes/ doughnuts/ sweetened pies/ baklawa/ basbosa/ konafa), fast food, and caffeinated drinks (coffee, tea), less than or equal to 2 times/week, while carbohydrates, fresh fruits and vegetables, legumes (fava beans/ hommos), dairy products unsweetened milk/ cheese/ yogurt, candies (sticky or not), crackers (biscuits/ chips), and chocolate were consumed 1–6 times/day by the majority of participants with dmft more than 4.

A statistically significant positive correlation was detected between dmft and legumes, sweetened milk, soft drinks, sweetened juice, and desserts (cakes/ doughnuts/ sweetened pies/ baklawa/ basbosa/ konafa), while a statistically significant negative correlation was detected between dmft and meat/poultry/fish, fresh fruits and vegetables, and dairy products (unsweetened milk/ cheese/ yogurt).

After adjusting the effect of age and gender, the following items were found to be significantly associated with high dmft: legumes, sweetened milk, soft drinks, sweetened juice, granulated sugars, and desserts (cakes/ doughnuts/ sweetened pies/ baklawa/ basbosa/ konafa). Conversely, meat/poultry/fish, fresh fruits and vegetables, and dairy products, including unsweetened milk/ cheese/ yogurt, were significantly associated with low dmft (Table 5).

**Table 5:** Correlation between dmft index and frequency of intake of different food items (Total number 105).

**Dietary habits**											
**Parameter and categories**		**Number (%)**			**M-W test**	**Correlation**		**Logistic regression**			
		**dmft = 0**	**dmft = 1–3**	**dmft** **>** **4**	***p*** **-value**	**rho**	***p*** **-value**	***p*** **-value**	OR	**95%CI for OR**	
		**7 (6.7%)**	**21 (20 %)**	**77 (73.3%)**						**Lower**	**Upper**
a. **Meat/poultry/fish**	**≤2 times/week 37 (35.2%)**	0	3 (8.1%)	34 (91.9%)	<0.05^*^	−0.415	<0.05^*^	<0.05^*^	0.269	0.141	0.515
	**3–6 times/week 33 (31.4%)**	1 (3.07%)	6 (18.2%)	26 (78.8%)							
	**1–6 times/day 35 (33.3%)**	6 (17.1%)	12 (34.3%)	17 (48.6%)							
b. **Carbohydrates**	**≤2 times/week 4 (3.8%)**	0	1 (25%)	3 (75%)	0.094	−0.110	0.264	0.359	0.623	0.227	1.713
	**3–6 times/week 14 (13.3%)**	0	2 (14.3%)	12 (85.7%)							
	**1–6 times/day 87 (82.8%)**	7 (8.1%)	18 (20.7%)	62 (71.3%)							
c. **Fresh fruits and vegetables**	**≤2 times/week 19 (18%)**	0	1 (5.3%)	18 (94.7%)	0.010^*^	−0.378	<0.05^*^	0.001^*^	0.197	0.073	0.536
	**3–6 times/week 25 (23.8%)**	0	2 (8%)	23 (92%)							
	**1–6 times/day 61 (58.1%)**	7 (11.5%)	18 (29.5%)	36 (59.0%)							
d. **Legumes (fava beans/ hommos)**	**≤2 times/week 47 (44.8%)**	5 (10.6%)	12 (25.5%)	30 (63.8%)	<0.05^*^	0.219	0.025^*^	0.029^*^	1.775	1.062	2.966
	**3–6 times/week 20 (19%)**	2 (10%)	3 (15%)	15 (75%)							
	**1–6 times/day 38 (36.2%)**	0	6 (15.79%)	32 (84.2%)							
e. **Sweetened cereals/ belela**	**≤2 times/week 62 (59%)**	2 (3.2%)	11 (17.7%)	49 (79.0%)	<0.05^*^	−0.145	0.140	0.180	0.714	0.436	1.168
	**3–6 times/week 17 (16.2%)**	2 (11.8%)	5 (29.4%)	10 (58.8%)							
	**1–6 times/day 26 (24.8%)**	3 (11.5%)	5 (19.2%)	18 (69.2%)							
f. **Dairy products un sweetened milk/ cheese/ yoghurt**	**≤2 times/week 23 (21.9%)**	1 (4.3%)	2 (8.7%)	20 (87%)	0.006^*^	−0.236	0.015^*^	0.025^*^	0.475	0.247	0.911
	**3–6 times/week 21 (20%)**	1 (4.7%)	2 (9.5%)	18 (85.7%)							
	**1–6 times/day 61(58.1%)**	5 (8.19%)	17 (27.86%)	39 (63.93%)							
g. **Sweetened milk**	**≤2 times/week 58 (55.2%)**	7 (12.1%)	16 (27.6%)	35 (60.3%)	<0.05^*^	0.326	0.001^*^	0.003^*^	2.775	1.422	5.417
	**3-6 times/week 18 (17.1%)**	0	2 (11.1%)	16 (88.8%)							
	**1–6 times/day 29 (27.6%)**	0	3 (10.3%)	26 (89.7%)							
h. **Dairy ice cream**	**≤2 times/week 76 (72.4%)**	6 (7.9%)	15 (19.7%)	55 (72.4%)	<0.05^*^	0.059	0.547	0.434	1.299	0.674	2.506
	**3–6 times/week 16 (15.2%)**	1 (6.3%)	4 (25%)	11 (68.7%)							
	**1–6 times/day 13 (12.4%)**	0	2 (15.4%)	11 (84.6%)							
i. **Soft drinks**	**≤2 times/week 67 (63.8%)**	7 (10.5%)	18 (26.9%)	42 (62.7%)	<0.05^*^	0.330	0.001^*^	0.004^*^	3.770	1.531	9.285
	**3–6 times/week 15 (14.3%)**	0	2 (13.3%)	13 (86.7%)							
	**1–6 times/day 23 (21.9%)**	0	1 (4.4%)	22 (95.6%)							
j. **Sweetened juice**	**≤2 times/week 46 (43.8%)**	3 (6.5%)	14 (30.4%)	29 (63.0%)	<0.05^*^	0.237	0.015^*^	0.016^*^	2.029	1.142	3.606
	**3–6 times/week 27 (25.7%)**	2 (7.4%)	6 (22.2%)	19 (70.4%)							
	**1–6 times/day 32 (30.5%)**	2 (6.3%)	1 (3.1%)	29 (90.6%)							
k. **Granulated sugars**	**≤2 times/week 72 (68.6%)**	5 (6.9%)	18 (25%)	49 (68.1%)	<0.05^*^	0.188	0.055	0.047^*^	1.943	1.008	3.744
	**3–6 times/week 10(9.5%)**	2 (20%)	1 (10%)	7 (70%)							
	**1–6 times/day 23 (21.9%)**	0	2 (8.70%)	21 (91.30%)							
l. **Candies (sticky or not)**	**≤2 times/week 25 (23.8%)**	1 (4%)	8 (32%)	16 (64%)	0.003^*^	0.162	0.099	0.125	1.470	0.899	2.404
	**3–6 times/week 20 (19%)**	3 (15%)	4 (20%)	13 (65%)							
	**1–6 times/day 60 (57.1%)**	3 (5%)	9 (15%)	48 (80%)							
m. **Halawa/ honey**	**≤2 times/week 54 (51.4%)**	4 (7.4%)	11 (20.4%)	39 (72.2%)	<0.05^*^	0.098	0.320	0.239	1.368	0.812	2.304
	**3–6 times/week 21 (20%)**	2 (9.5%)	7 (33.3%)	12 (57.2%)							
	**1–6 times/day 30 (28.6%)**	1 (3.3%)	3 (10%)	26 (86.7%)							
n. **Crackers (biscuits/ chips)**	**≤2 times/week 24 (22.9%)**	1 (4.2%)	4 (16.7%)	19 (79.2%)	0.023^*^	0.020	0.839	0.972	0.991	0.593	1.657
	**3–6 times/week 16 (15.2%)**	2 (12.5%)	5 (31.3%)	9 (56.3%)							
	**1–6 times/day 65 (61.9%)**	4 (6.2%)	12 (18.5%)	49 (75.4%)							
o. **Cakes/ doughnuts/ sweetened pies/ baklawa/ basbosa/ konafa**	**≤2 times/week 70 (66.7%)**	6 (8.6%)	19 (27.1%)	45 (64.3%)	<0.05^*^	0.290	0.003^*^	0.009^*^	3.299	1.346	8.085
	**3–6 times/week 15 (14.3%)**	1 (6.7%)	1 (6.6%)	13 (86.7%)							
	**1–6 times/day 20 (19%)**	0	1 (5.0%)	19 (95.0%)							
p. **Fast food**	**≤2 times/week 76 (72.4%)**	7 (9.2%)	16 (21.1%)	53 (69.7%)	<0.05^*^	0.158	0.108	0.100	1.940	.881	4.273
	**3–6 times/week 17 (16.2%)**	0	4 (23.5%)	13 (76.5%)							
	**1–6 times/day 12 (11.4%)**	0	1 (8.3%)	11 (91.7%)							
q. **Chocolate**	**≤2 times/week 41 (39%)**	1 (2.4%)	8 (19.5%)	32 (78.1%)	<0.05^*^	−0.028	0.775	0.748	0.926	0.577	1.485
	**3–6 times/week 18 (171%)**	2 (11.1%)	6 (33.3%)	10 (55.6%)							
	**1–6 times/day 46 (43.8%)**	4 (8.7%)	7 (15.2%)	35 (76.1%)							
r. **Caffeinated drinks (coffee, tea)**	**≤2 times/week 72 (68.6%)**	7 (9.7%)	16 (22.2%)	49 (68.1%)	<0.05^*^	0.180	0.066	0.095	1.669	0.914	3.048
	**3–6 times/week 10 (9.5%)**	0	1 (10%)	9 (90.0%)							
	**1–6 times/day 23 (21.9%)**	0	4 (17.4%)	19 (82.6%)							

* Statistically significant.

#### Correlation between deft index and different risk factors

Most of the studied population 145 (54.9%) had a deft index of more than 4. The highest percentage of participants with deft index of more than 4 fell into the AII age group. The highest percentage of participants with deft index of more than 4 were females. The highest percentage of participants with deft index of more than 4 were overweight or obese. A statistically significant negative correlation was detected between age and deft also between deft and healthy lifestyle habits. A statistically significant positive correlation was detected between deft and BMI (Table 6).

**Table 6:** Correlation between deft index and different risk factors (Total number 264).

**Parameter and categories**		**Number (%)**			**M-W test**	**Correlation**		**Logistic regression**			
		**deft = 0 33 (12.5%)**	**deft = 1-3** **86 (32.57%)**	**deft >4 145 (54.92%)**	***p*-value**	**rho**	***p*-value**	***p*-value**	**OR**	**95%CI for OR**	
										**Lower**	**Upper**
**Age (years)**	**AI 67 (25.4%)**	5 (7.5%)	16 (23.9%)	46 (68.6%)	<0.05^*^	−0.278	<0.05^*^				
	**AII 97 (36.7%)**	7 (7.2%)	28 (28.9%)	62 (63.9%)							
	**AIII 100 (37.9%)**	21 (21.0%)	42 (42.0%)	37 (37.0%)							
**Gender**	**Boys 124 (47.0%)**	17 (13.7%)	41 (33.1%)	66 (53.2%)	<0.05^*^	0.037	0.549				
	**Girls 140 (53.0%)**	16 (11.4%)	45 (32.1%)	79 (56.5%)							
**BMI**	**Normal (≥5- <85) 88 (33.3%)**	16 (18.9%)	36 (40.9%)	36 (40.1%)	<0.05^*^	0.203	0.001^*^				
	**Overweight (≥85- <95) and**	17 (9.7%)	50 (28.4%)	109 (61.9%)							
	**Obese (≥95) 176 (66.7%)**
**Lifestyle habits**
**Lifestyle habits**	**Unhealthy habits 108 (40.9%)**	6 (5.6%)	37 (34.3%)	65 (60.1%)	<0.05^*^	−0.173	0.005^*^				
	**Moderate 140 (53%)**	23 (16.4%)	42 (30%)	75 (53.1%)							
	**Healthy habits 16 (6.1%)**	4 (25%)	7 (43.7%)	5 (31.3%)							

* Statistically significant.

The highest percentage of participants with deft index of more than 4 consumed meat/poultry/fish, sweetened cereals/ belela, dairy ice cream, soft drinks, sweetened juice, granulated sugars, halawa/ honey, desserts (cakes/ doughnuts/ sweetened pies/ baklawa/ basbosa/ konafa), fast food, chocolate, and caffeinated drinks less than or equal to 2 times/week, while carbohydrates, fresh fruits and vegetables, dairy products, sweetened milk, candies, and crackers were consumed 1 to 6 times daily.

A statistically significant positive correlation was detected between deft and sweetened milk, dairy ice cream, sweetened juice, candies, and crackers. In contrast, a statistically significant negative correlation was detected between deft and meat/poultry/fish, fresh fruits, and vegetables.

After adjusting the effect of age and gender, the following items were found to be significantly associated with high deft, sweetened milk, dairy ice cream, soft drinks, candies, and crackers. On the contrary, meat/poultry/fish, fresh fruits, and vegetables were associated with low deft (Table 7).

**Table 7:** Correlation between deft index and frequency of intake of different food items (Total number 264).

**Dietary habits**											
		**Number (%)**			**M-W test**	**Correlation**		**Logistic regression**			
		**deft = 0**	**deft = 1–3**	**deft** **>** **4**	***p*** **-value**	**rho**	***p*** **-value**	***p*** **-value**	**OR**	**95%CI for OR**	
		**33 (12.5%)**	**86 (32.57%)**	**145 (54.92%)**						**Lower**	**Upper**
a. Meat/poultry/fish	≤ 2 times/week 93 (35.2%)	6 (6.4%)	29 (31.2%)	58 (62.4%)	<0.05^*^	−0.186	0.002^*^	<0.05^*^	0.584	0.429	0.795
	3–6 times/week 94 (35.6%)	11 (11.7%)	28 (29.8%)	55 (58.5%)							
	1–6 times/day 77 (29.2%)	16 (20.8%)	29 (37.6%)	32 (41.6%)							
b. Carbohydrates	≤ 2 times/week 22 (8.3%)	6 (27.3%)	6 (27.3%)	10 (45.4%)	0.001^*^	0.002	0.973	0.983	0.996	0.680	1.458
	3–6 times/week 59 (22.4%)	3 (5.1%)	21 (35.6%)	35 (59.3%)							
	1–6 times/day 183 (69.3%)	24 (13.2%)	59 (32.2%)	100 (54.6%)							
c. Fresh fruits and vegetables	≤ 2 times/week 77 (29.2%)	7 (9.1%)	21 (27.3%)	49 (63.7%)	0.010^*^	−0.172	0.005^*^	0.002^*^	0.632	0.472	0.846
	3–6 times/week 55 (20.8%)	2 (3.6%)	19 (34.6%)	34 (61.8%)							
	1–6 times/day 132 (50%)	24 (18.2%)	46 (34.8%)	62 (47%)							
d. Legumes (fava beans/ hommos)	≤ 2 times/week 117 (44.3%)	18 (15.4%)	41 (35.0%)	58 (49.6%)	<0.05^*^	0.110	0.073	0.081	1.274	0.971	1.672
	3–6 times/week 52 (19.7%)	6 (11.5%)	17 (32.7%)	29 (55.8%)							
	1–6 times/day 95 (36%)	9 (9.5%)	28 (29.5%)	58 (61.0%)							
e. Sweetened cereals/ belela	≤ 2 times/week 167 (63.3%)	21 (12.6%)	55 (32.9%)	91 (54.5%)	<0.05^*^	0.022	0.726	0.485	1.103	0.837	1.453
	3–6 times/week 27 (10.3%)	4 (14.8%)	10 (37.0%)	13 (48.2%)							
	1–6 times/day 70 (26.5%)	8 (11.4%)	21 (30.0%)	41 (58.6%)							
f. Dairy products un sweetened milk/ cheese/ yogurt	≤ 2 times/week 62 (23.4%)	4 (6.5%)	23 (37.1%)	35 (56.5%)	0.828	−0.073	0.237	0.105	0.786	0.588	1.051
	3–6 times/week 45 (17.1%)	4 (8.9%)	14 (31.1%)	27 (60.0 %)							
	1–6 times/day 157 (59.5%)	25 (15.9%)	49 (31.2%)	83 (52.9%)							
g. Sweetened milk	≤ 2 times/week 125 (47.4%)	24 (19.2%)	43 (34.4%)	58 (46.4%)	<0.05^*^	0.185	0.003^*^	0.005^*^	1.468	1.126	1.913
	3–6 times/week 36 (13.6%)	4 (11.1%)	9 (25.0%)	23 (63.9%)							
	1–6 times/day 103 (39.0%)	5 (4.9%)	34 (33.0%)	64 (62.1%)							
h. Dairy ice cream	≤ 2 times/week 158 (59.9%)	24 (15.2%)	58 (36.7%)	76 (48.1%)	<0.05^*^	0.182	0.003^*^	0.005^*^	1.586	1.149	2.191
	3–6 times/week 55 (20.8%)	6 (10.9%)	16 (29.1%)	33 (60.0%)							
	1–6 times/day 51 (19.39%)	3 (5.9%)	12 (23.5%)	36 (70.6%)							
i. Soft drinks	≤ 2 times/week 176 (66.97%)	25 (14.2%)	60 (34.1%)	91 (51.7%)	<0.05^*^	0.118	0.055	0.003^*^	1.617	1.172	2.232
	3–6 times/week 35 (13.3%)	4 (11.4%)	14 (40.0%)	17 (48.6%)							
	1–6 times/day 53 (20%)	4 (7.6%)	12 (22.6%)	37 (69.8%)							
j. Sweetened juice	≤ 2 times/week 129 (48.9%)	21 (16.3%)	47 (36.4%)	61 (47.3%)	<0.05^*^	0.122	0.048^*^	0.075	1.297	0.974	1.728
	3–6 times/week 59 (22.4%)	3 (5.1%)	16 (27.1%)	40 (67.8%)							
	1–6 times/day 76 (28.8%)	9 (11.8%)	23 (30.3%)	44 (57.9%)							
k. Granulated sugars	≤ 2 times/week 198 (75 %)	26 (13.1%)	69 (34.9%)	103 (52.0%)	<0.05^*^	0.096	0.120	0.096	1.317	0.952	1.821
	3–6 times/week 18 (6.8%)	2 (11.1%)	5 (27.8%)	11 (61.1%)							
	1–6 times/day 48 (18.2%)	5 (10.4%)	12 (25.0%)	31 (64.6%)							
l. Candies (sticky or not)	≤ 2 times/week 98 (37.1%)	13 (13.3%)	40 (40.8%)	45 (45.9%)	<0.05^*^	0.141	0.021^*^	0.031^*^	1.340	1.027	1.750
	3–6 times/week 47 (17.8%)	7 (14.9%)	15 (31.2%)	25 (53.2%)							
	1–6 times/day 119 (45.1%)	13 (10.9%)	31 (26.1%)	75 (63.0 %)							
m. Halawa/ honey	≤ 2 times/week 148 (56.1%)	20 (13.5%)	48 (32.4%)	80 (54.1%)	<0.05^*^	0.013	0.833	0.844	0.973	0.744	1.273
	3–6 times/week 39 (14.8%)	6 (15.4%)	8 (20.5%)	25 (64.1%)							
	1–6 times/day 77 (29.1%)	7 (9.1%)	30 (39%)	40 (51.9%)							
n. Crackers (biscuits/ chips)	≤ 2 times/week 56 (21.2%)	7 (12.5%)	24 (42.9%)	25 (44.6%)	0.702	0.141	0.022^*^	0.035^*^	1.364	1.022	1.821
	3–6 times/week 46 (17.4%)	8 (17.4%)	17 (37%)	21 (45.6%)							
	1–6 times/day 162 (61.4%)	18 (11.1%)	45 (27.8%)	99 (61.1%)							
o. Cakes/ doughnuts/ sweetened pies/ baklawa/ basbosa/ konafa	≤ 2 times/week 180 (68.2%)	23 (12.8%)	60 (33.3%)	97 (53.9%)	<0.05^*^	0.051	0.411	0.165	1.285	0.902	1.830
	3–6 times/week 51 (19.3%)	6 (11.7%)	21 (41.2%)	24 (47.1%)							
	1–6 times/day 33 (12.5%)	4 (12.1%)	5 (15.2%)	24 (72.7%)							
p. Fast food	≤ 2 times/week 168 (63.6%)	20 (11.9%)	58 (34.5%)	90 (53.6%)	<0.05^*^	0.026	0.670	0.315	1.178	0.856	1.622
	3–6 times/week 50 (18.9%)	5 (10.0%)	18 (36.0%)	27 (54.0%)							
	1–6 times/day 46 (17.4%)	8 (17.4%)	10 (21.7%)	28 (60.9%)							
q. Chocolate	≤ 2 times/week 122 (46.2%)	14 (11.5%)	47 (38.5%)	61 (50.0%)	<0.05^*^	0.063	0.308	0.531	1.092	0.829	1.438
	3–6 times/week 52 (19.7%)	5 (9.6%)	17 (32.7%)	30 (57.7%)							
	1–6 times/day 90 (34.1%)	14 (15.6%)	22 (24.4%)	54 (60.0%)							
r. Caffeinated drinks (coffee, tea)	≤ 2 times/week 196 (74.2%)	25 (12.8%)	68 (34.7%)	103 (52.5%)	<0.05^*^	0.084	0.175	0.069	1.348	0.978	1.858
	3–6 times/week 21 (8%)	5 (23.8%)	5 (23.8%)	11 (52.4%)							
	1–6 times/day 47 (17.8%)	3 (6.4%)	13 (27.7%)	31 (65.9%)							

* Statistically significant.

#### Correlation between dmft index and different risk factors

The majority of participants 173 (65.5%) had DMFT = 0. The highest percentage of participants with DMFT index of more than 4 fell into the AIII age group. The highest percentage of participants with DMFT index of more than 4 were females. The highest percentage of participants with DMFT index of more than 4 were overweight or obese. A statistically significant positive correlation was detected between age and DMFT, while a non-significant positive correlation was detected between gender, BMI, and DMFT (Table 8).

**Table 8:** Correlation between DMFT index and different risk factors (Total number 264).

**Parameter and categories**		**Number (%)**			**M-W test**	**Correlation**		**Logistic regression**			
		**DMFT = 0 173 (65.53%)**	**DMFT = 1–3** **54 (20.45%)**	**DMFT > 4 37 (14.01%)**	***p*-value**	**rho**	***p*-value**	***p*-value**	**OR**	**95% CI for OR**	
										**Lower**	**Upper**
Age (years)	AI 67 (25.4%)	63 (94.03%)	4 (5.97%)	0	<0.05^*^	0.460	<0.05^*^				
	AII 97 (36.7%)	70 (72.16%)	17 (17.53%)	10 (10.31%)							
	AIII 100 (37.9%)	40 (40%)	33 (33%)	27 (27%)							
Gender	Boys 124 (47%)	85 (68.55%)	24 (19.35%)	15 (12.10%)	0.012^*^	0.063	0.307				
	Girls 140 (53%)	88 (62.86%)	30 (21.43%)	22 (15.71%)							
BMI	Normal (≥5– <85) 88 (33.3%)	62 (70.45%)	16 (18.18%)	10 (11.36%)	<0.05^*^	0.075	0.225				
	Overweight (≥85– <95) and	111 (63.07%)	38 (21.59%)	27 (15.34%)							
	Obese (≥ 95) 176 (66.7%)
**Lifestyle habits**
Lifestyle habits	Unhealthy habits 108 (40.9%)	73 (67.6%)	15 (13.9%)	20 (18.5%)	<0.05^*^	−0.009	0.883				
	Moderate 140 (53%)	88 (62.8%)	37 (26.4%)	15 (10.7%)							
	Healthy habits 16 (6.1%)	12 (75%)	2 (12.5%)	2 (12.5%)							

* Statistically significant.

The highest percentage of participants with DMFT more than 4 consumed meat/poultry/fish, legumes, sweetened cereals, dairy ice cream, soft drinks, granulated sugars, desserts (cakes/ doughnuts/ sweetened pies/ baklawa/ basbosa/ konafa), fast food, and caffeinated drinks less than or equal to 2 times/week, while carbohydrates, fresh fruits and vegetables, dairy products, sweetened juice, candies, halawa/ honey, crackers, and chocolate were consumed 1–6 times/day.

A statistically significant positive correlation was detected between DMFT and soft drinks, sweetened juice, and desserts (cakes/ doughnuts/ sweetened pies/ baklawa/ basbosa/ konafa), while a statistically significant negative correlation was detected between DMFT and fresh fruits and vegetables.

After adjusting the effect of age and gender, the following items were found to be significantly associated with high DMFT: soft drinks, sweetened juice, and desserts (cakes/ doughnuts/ sweetened pies/ baklawa/ basbosa/ konafa). In contrast, fresh fruits and vegetables were significantly associated with low DMFT (Table 9).

**Table 9:** Correlation between DMFT index and frequency of intake of different food items (Total number 264).

**Dietary habits**											
**Parameter and categories**		**Number (%)**			**M-W test**	**Correlation**		**Logistic regression**			
		**DMFT = 0**	**DMFT = 1–3**	**DMFT** **>** **4**	***p*** **-value**	**rho**	***p*** **-value**	***p*** **-value**	**OR**	**95%CI for OR**	
		**173 (65.53%)**	**54 (20.45%)**	**37 (14.01%)**						**Lower**	**Upper**
a. Meat/poultry/fish	≤ 2 times/week 93 (35.2%)	55 (59.1%)	24 (25.8%)	14 (15.1%)	<0.05^*^	−0.110	0.074	0.072	0.748	0.545	1.027
	3-6 times/week 94 (35.6%)	61 (64.9%)	20 (21.3%)	13 (13.8%)							
	1-6 times/day 77 (29.2%)	57 (74%)	10 (13%)	10 (13%)							
b. Carbohydrates	≤ 2 times/week 22 (8.3%)	13 (59.1%)	6 (27.3%)	3 (13.6%)	<0.05^*^	−0.058	0.349	0.368	0.841	0.577	1.226
	3-6 times/week 59 (22.4%)	36 (61.0%)	15 (25.4%)	8 (13.6%)							
	1-6 times/day 183 (69.3%)	124 (67.8%)	33 (18.0%)	26 (14.2%)							
c. Fresh fruits and vegetables	≤ 2 times/week 77 (29.2%)	45 (58.4%)	18 (23.4%)	14 (18.2%)	<0.05^*^	−0.128	0.038^*^	0.041^*^	0.741	0.557	0.987
	3-6 times/week 55 20.8%)	33 (60.0%)	15 (27.3%)	7 (12.7%)							
	1-6 times/day 132 (50%)	95 (72%)	21 (15.9%)	16 (12.1%)							
d. Legumes (fava beans/ hommos)	≤ 2 times/week 117 (44.3%)	78 (66.7%)	22 (18.8%)	17 (14.5%)	<0.05^*^	−0.050	0.414	0.367	0.879	0.664	1.163
	3–6 times/week 52 (19.7%)	26 (50.0%)	15 (28.9%)	11 (21.1%)							
	1–6 times/day 95 (34.8%)	69 (72.6%)	17 (17.9%)	9 (9.5%)							
e. Sweetened cereals/ belela	≤ 2 times/week 167 (63.3%)	110 (65.9%)	39 (23.3%)	18 (10.8%)	0.143	0.061	0.322	0.228	1.191	0.896	1.582
	3–6 times/week 27 (10.2%)	21 (77.8%)	4 (14.8%)	2 (7.4%)							
	1–6 times/day 70 (26.5 %)	42 (60.0%)	11 (15.7%)	17 (24.3%)							
f. Dairy products un sweetened milk/ cheese/ yogurt	≤ 2 times/week 62 (23.5%)	41 (66.1%)	9 (14.5%)	12 (19.4%)	<0.05^*^	−0.079	0.200	0.274	0.847	0.629	1.140
	3-6 times/week 45 (17.1%)	23 (51.1%)	16 (35.6%)	6 (13.3%)							
	1-6 times/day 157 (59.4%)	109 (69.4%)	29 (18.5%)	19 (12.1%)							
g. Sweetened milk	≤ 2 times/week 125 (47.4%)	84 (67.2%)	25 (20.0%)	16 (12.8%)	<0.05^*^	0.062	0.316	0.301	1.151	0.881	1.504
	3-6 times/week 36 (13.6%)	27 (75.0%)	4 (11.1%)	5 (13.9%)							
	1-6 times/day 103 (39.0%)	62 (60.2%)	25 (24.3%)	16 (15.5%)							
h. Dairy ice cream	≤ 2 times/week158 (59.9%)	103 (65.2%)	34 (21.2%)	21 (13.3%)	0.121	−0.017	0.783	0.668	0.933	0.680	1.281
	3–6 times/week 55 (20.8%)	34 (61.8%)	11 (20.0%)	10 (18.2%)							
	1–6 times/day 51 (19.3%)	36 (70.6%)	9 (17.7%)	6 (11.7%)							
i. Soft drinks	≤ 2 times/week 176 (66.7%)	122 (69.3%)	35 (19.9%)	19 (10.8%)	0.792	0.139	0.024^*^	0.018^*^	1.440	1.065	1.948
	3–6 times/week 35 (13.2%)	22 (62.9%)	9 (25.7%)	4 (11.4%)							
	1–6 times/day 53 (20.1%)	29 (54.7%)	10 (18.9%)	14 (26.4%)							
j. Sweetened juice	≤ 2 times/week 129 (48.8%)	95 (73.6%)	23 (17.8%)	11 (8.5%)	<0.05^*^	0.167	0.007^*^	0.008^*^	1.479	1.106	1.980
	3–6 times/week 59 (22.4%)	33 (55.9%)	17 (28.8%)	9 (15.3%)							
	1–6 times/day 76 (28.8%)	45 (59.2%)	14 (18.4%)	17 (22.4%)							
k. Granulated sugars	≤ 2 times/week 198 (75%)	137 (69.2%)	35 (17.7%)	26 (13.1%)	0.097	0.101	0.103	0.261	1.193	0.877	1.622
	3–6 times/week 18 (6.8%)	5 (27.8%)	8 (44.4%)	5 (27.8%)							
	1–6 times/day 48 (18.2%)	31 (64.6%)	11 (22.9%)	6 (12.5%)							
l. Candies (sticky or not)	≤ 2 times/week 98 (37.1%)	69 (70.4%)	16 (16.3%)	13 (13.3%)	<0.05^*^	0.091	0.139	0.138	1.237	0.934	1.639
	3–6 times/week 47 (17.8%)	31 (65.9%)	14 (29.8%)	2 (4.3%)							
	1–6 times/day 119 (45.1%)	73 (61.3%)	24 (20.2%)	22 (18.5%)							
m. Halawa/ honey	≤ 2 times/week 148 (56.1%)	101 (68.3%)	34 (22.9%)	13 (8.8%)	0.002^*^	0.094	0.128	0.132	1.240	0.937	1.642
	3-6 times/week 39 (14.8%)	24 (61.5%)	8 (20.5%)	7 (18.0%)							
	1-6 times/day 77 (29.1%)	48 (62.3%)	12 (15.6%)	17 (22.1%)							
n. Crackers (biscuits/ chips)	≤ 2 times/week 56 (21.2%)	40 (71.4%)	10 (17.9%)	6 (10.7%)	<0.05^*^	0.043	0.488	0.413	1.139	0.834	1.554
	3-6 times/week 46 (17.4%)	28 (60.9%)	12 (26.1%)	6 (13.0%)							
	1-6 times/day 162 (61.4%)	105 (64.8%)	32 (19.8%)	25 (15.4%)							
o. Cakes/ doughnuts/ sweetened pies/ baklawa/ basbosa/ konafa	≤ 2 times/week 180 (68.2%)	125 (69.5%)	40 (22.2%)	15 (8.3%)	0.503	0.158	0.010^*^	0.015^*^	1.538	1.089	2.172
	3–6 times/week 51 (19.3%)	29 (56.8%)	9 (17.7%)	13 (25.5%)							
	1–6 times/day 33 (12.5%)	19 (57.6%)	5 (15.1%)	9 (27.3%)							
p. Fast food	≤ 2 times/week 168 (63.6%)	115 (68.45%)	36 (21.4%)	17 (10.1%)	0.511	0.102	0.097	0.114	1.291	0.941	1.772
	3-6 times/week 50 (19%)	30 (60.0%)	10 (20.0%)	10 (20.0%)							
	1-6 times/day 46 (17.4%)	28 (60.9%)	8 (17.4%)	10 (21.7%)							
q. Chocolate	≤ 2 times/week 122 (46.2%)	78 (63.9%)	29 (23.8%)	15 (12.3%)	<0.05^*^	−0.001	0.981	0.997	1.001	0.754	1.328
	3–6 times/week 52 (19.7%)	35 (67.3%)	13 (25.0%)	4 (7.8%)							
	1–6 times/day 90 (34.1%)	60 (66.7%)	12 (13.3%)	18 (20.0%)							
r. Caffeinated drinks (coffee, tea)	≤ 2 times/week 196 (74.2%)	130 (66.3%)	38 (19.4%)	28 (14.3%)	0.128	0.014	0.826	0.943	1.012	0.737	1.388
	3–6 times/week 21 (8%)	12 (57.1%)	5 (23.8%)	4 (19.1%)							
	1–6 times/day 47 (17.8%)	31 (66%)	11 (23.4%)	5 (10.6%)							

* Statistically significant.

### Correlation between BMI and different risk factors

Most of the studied population 247 (66.9%) were overweight (≥ 85– <95) and obese (≥ 95), while 122(33.1%) of the participants had normal BMI (≥ 5– <85). The highest percentage of overweight/obese participants fell into AII age group. The highest percentage of overweight/obese participants were males. Overweight/obese participants had the highest percentages of unhealthy lifestyle habits.

A non-statistically positive correlation was detected between BMI and age. Moreover, a non-statistically negative correlation was detected between BMI and gender. On the contrary, a statistically significant negative correlation was detected between BMI and healthy lifestyle habits (Table 10).

**Table 10:** Correlation between BMI and different risk factors (Total number 369).

**Parameter and categories**		**Number (%)**		**M-W test**	**Correlation**		**Logistic regression**			
		**Normal (≥5– <85)**	**Over weight (≥85- <95) and Obese (≥95)**	***p*-value**	**rho**	***p*-value**	***p*-value**	**OR**	**95% CI for OR**	
									**Lower**	**Upper**
Age (years)	AI	60 (35.1%)	111 (64.9%)	<0.05^*^	0.033	0.524				
	AII	30 (30.6%)	68 (69.4%)							
	AIII	32 (32%)	68 (68%)							
Gender	Boys	58 (32.2%)	122 (67.8%)	<0.05^*^	−0.017	0.739				
	**Girls**	64 (33.9%)	125 (66.1%)							
**Lifestyle habits**
Lifestyle habits	Unhealthy habits	40 (26.32%)	112 (73.7%)	<0.05^*^	−0.146	0.005*				
	Moderate	67 (35.26%)	123 (64.7)							
	Healthy habits	15 (55.56%)	12 (44.4%)							

* Statistically significant.

The highest percentage of overweight/obese participants consumed meat/poultry/fish, carbohydrates, fresh fruits and vegetables, dairy products, and chocolate less than or equal to 2 times/week and consumed sweetened milk 3 to 6 times per week. Legumes, sweetened cereals/ belela, dairy ice cream, soft drinks, sweetened juice, granulated sugars, halawa/honey, crackers, desserts (cakes/ doughnuts/ sweetened pies/ baklawa/ basbosa/ konafa), fast food, and caffeinated drinks were consumed 1 to 6 times per day within overweight/obese participants, while candies were nearly equally consumed less than or equal to 2 times/week and 1–6 times/day.

A statistically significant positive correlation was detected between BMI and legumes, dairy ice cream, soft drinks, granulated sugars, desserts (cakes/ doughnuts/ sweetened pies/ baklawa/ basbosa/ konafa), fast food, and caffeinated drinks, while a statistically significant negative correlation was detected between BMI and meat/poultry/fish, fresh fruits, and vegetables.

After adjusting the effect of age and gender, the following items were found to be associated with high BMI: legumes, dairy ice cream, soft drinks, granulated sugars, desserts (cakes/ doughnuts/ sweetened pies/ baklawa/ basbosa/ konafa), fast food, and caffeinated drinks. While meat/poultry/fish, fresh fruits, and vegetables were significantly associated with low BMI (Table 11).

**Table 11:** Correlation between BMI and frequency of intake of different food items (Total number 369).

**Dietary habits**										
**Parameter and categories**		**Number (%)**		**M-W test**	**Correlation**		**Logistic regression**			
		**Normal**	**Over weight**	***p*** **-value**	**rho**	***p*** **-value**	***p*** **-value**	**OR**	**95%CI for OR**	
		**(≥5- <85)**	**(≥85- <95) and Obese (≥95)**						**Lower**	**Upper**
a. Meat/poultry/fish	≤ 2 times/week	25 (19.2%)	105 (80.8%)	<0.05^*^	−0.270	<0.05^*^	<0.05^*^	0.478	0.358	0.637
	3–6 times/week	40 (31.5%)	87 (68.5%)							
	1–6 times/day	57 (50.9%)	55 (49.1%)							
b. Carbohydrates	≤ 2 times/week	7 (26.9%)	19 (73.1%)	0.251	0.046	0.383	0.548	1.116	.779	1.600
	3–6 times/week	30 (41.1%)	43 (58.9%)							
	1–6 times/day	85 (31.5%)	185 (68.5%)							
c. Fresh fruits and vegetables	≤ 2 times/week	6 (6.3%)	90 (93.7%)	<0.05^*^	−0.395	<0.05^*^	<0.05^*^	.280	.195	.403
	3–6 times/week	20 (25%)	60 (75%)							
	1–6 times/day	96 (49.7%)	97 (50.3%)							
d. Legumes (fava beans/ hommos)	≤ 2 times/week	65 (39.6%)	99 (60.4%)	<0.05^*^	0.164	0.002^*^	0.002^*^	1.502	1.167	1.931
	3–6 times/week	28 (38.9%)	44 (61.1%)							
	1–6 times/day	29 (21.8%)	104 (78.2%)							
e. Sweetened cereals/ belela	≤ 2 times/week	80 (34.9%)	149 (65.1%)	<0.05^*^	0.084	0.107	0.051	1.299	.998	1.689
	3–6 times/week	21 (47.7%)	23 (52.3%)							
	1–6 times/day	21 (21.9%)	75 (78.1%)							
f. Dairy products unsweetened milk/ cheese/ yoghurt	≤ 2 times/week	26 (30.6%)	59 (69.4%)	<0.05^*^	−0.035	0.500	0.537	.920	.706	1.199
	3–6 times/week	21 (31.8%)	45 (68.2%)							
	1–6 times/day	75 (34.4%)	143 (65.6%)							
g. Sweetened milk	≤ 2 times/week	70 (38.3%)	113 (61.7%)	<0.05^*^	0.092	0.076	0.094	1.231	0.966	1.568
	3–6 times/week	13 (24.1%)	41 (75.9%)							
	1–6 times/day	39 (29.5%)	93 (70.5%)							
h. Dairy ice cream	≤ 2 times/week	88 (37.6%)	146 (62.4%)	<0.05^*^	0.142	0.006^*^	0.005^*^	1.564	1.147	2.133
	3–6 times/week	22 (30.9%)	49 (69.0%)							
	1–6 times/day	12 (18.7%)	52 (81.3%)							
i. Soft drinks	≤ 2 times/week 67 (63.8%)	98 (40.3%)	145 (59.7%)	<0.05^*^	0.265	<0.05^*^	<0.05^*^	2.700	1.862	3.916
	3–6 times/week 15 (14.3%)	23 (46%)	27 (54%)							
	1–6 times/day	1 (1.3%)	75 (98.7%)							
j. Sweetened juice	≤ 2 times/week	64 (36.6%)	111 (63.4%)	<0.05^*^	0.079	0.129	0.594	1.306	0.944	1.583
	3–6 times/week	28 (32.6%)	58 (67.4%)							
	1–6 times/day	30 (27.8%)	78 (72.2%)							
h. Granulated sugars	≤ 2 times/week	99 (36.7%)	171 (63.3%)	<0.05^*^	0.148	0.004^*^	0.001^*^	1.675	1.221	2.299
	3–6 times/week	13 (46.4%)	15 (53.6%)							
	1–6 times/day	10 (14.1%)	61 (85.9%)							
i. Candies (sticky or not)	≤ 2 times/week	39 (31.7%)	84 (68.3%)	<0.05^*^	0.006	0.901	0.902	1.016	0.795	1.297
	3–6 times/week	26 (38.8%)	41 (61.2%)							
	1–6 times/day	57 (31.8%)	122 (68.2%)							
j. Halawa/ honey	≤ 2 times/week	66 (32.7%)	136 (67.3%)	<0.05^*^	0.021	0.690	0.544	1.080	0.842	1.386
	3–6 times/week	26 (43.3%)	34 (56.7%)							
	1–6 times/day	30 (28.0%)	77 (72%)							
k. Crackers (biscuits/ chips)	≤ 2 times/week	26 (32.5%)	54 (67.5%)	<0.05^*^	0.044	0.405	0.517	1.091	0.839	1.419
	3–6 times/week	26 (41.9%)	36 (58.1%)							
	1–6 times/day	70 (30.8%)	157 (69.2%)							
l. Doughnuts/ sweetened pies/ baklawa/ basbosa/ konafa	≤ 2 times/week	91 (36.4%)	159 (63.6%)	<0.05^*^	0.108	0.038^*^	0.039^*^	1.399	1.017	1.925
	3–6 times/week	19 (28.8%)	47 (71.2%)							
	1–6 times/day	12 (22.6%)	41 (77.4%)							
m. Fast food	≤ 2 times/week	94 (38.5%)	150 (61.5%)	<0.05^*^	0.191	<0.05^*^	<0.05^*^	2.008	1.411	2.856
	3–6 times/week	23 (34.3%)	44 (65.7%)							
	1–6 times/day	5 (8.6%)	53 (91.4%)							
n. Chocolate	≤ 2 times/week	47 (28.8%)	116 (71.2%)	<0.05^*^	−0.035	0.502	0.587	.935	.733	1.192
	3–6 times/week	32 (45.7%)	38 (54.3%)							
	1–6 times/day	43 (31.6%)	93 (68.4%)							
o. Caffeinated drinks (coffee, tea)	≤ 2 times/week	102 (38.1%)	166 (61.9%)	<0.05^*^	0.178	0.001*	<0.05*	1.726	1.252	2.381
	3–6 times/week	8 (25.8%)	23 (74.2%)							
	1–6 times/day	12 (17.1%)	58 (82.9%)							

* Statistically significant.

## Discussion

Dental caries is considered the most prevalent non-communicable chronic disease worldwide, affecting all ages, from infants to old adults. It is a biofilm-mediated, sugar-induced, multifactorial, dynamic disease that leads to a cyclic demineralization and remineralization of dental hard tissues (80). This study examined the correlation between caries prevalence and malnutrition (overweight/obesity) in association with unhealthy food consumption patterns.

The strengths of our study include the collection of data representative to the whole nation since the patients come to the clinics of the Faculty of Dentistry, Cairo University from all the Egyptian governorates, and the high-quality outcome data. An additional strength is the use of a questionnaire validated by the WHO. Despite these strengths, the exposure measurement used in our study has several weaknesses including the sampling technique, where a non-random sampling technique was used as all the participants were recruited from the outpatient clinics of the Faculty of Dentistry, Cairo University. In addition, another major limitation is the cross-sectional study design, as the exposure and outcome are concurrently assessed. Finally, this study did not include questions about participants' oral hygiene and socioeconomic status which can be justified by the inclusion of such questions in a previous study among Egyptian adults (69) and children (47) conducted by our group. As per our previous study, poor oral hygiene was one of the risk factors significantly associated with caries prevalence among Egyptian adults (69) and children (47).

Study participants were recruited from the pediatrics dental clinics, Faculty of Dentistry, Cairo University. Hence, the majority of participants 342 (93.7%) suffered from dental caries, while only 27(7.3%) were caries-free. The highest mean dmft 6.9(± 4.6) and deft 5.5(± 3.9) were detected in an age group ranging from 5 to 6.9 years. This result could be attributed to lack of parental education, which affects their attitude regarding dental healthcare, with subsequent negligence toward maintaining a healthy primary dentition. Parents' ignorance can result in neglecting seeking dental treatment for their children as they regard primary teeth as unimportant and replaceable by permanent teeth (47, 81). On the contrary, the highest mean DMFT 1.8 (± 2.1) was detected in age group ranging from 9 to 10 years. A statistically significant positive correlation was detected between age and DMFT. Early loss of primary teeth due to a lack of parental education, with subsequent early eruption of their permanent successors, subjects permanent teeth to poor oral environment and to multiple caries-promoting factors (81).

The absence of dental services within the primary healthcare centers in Egypt leads to limited or no access to effective dental care (82). According to a WHO-sponsored epidemiological study on the state of oral health in Egypt, dental services were not being used to their full potential, and 40% of the included individuals stated that they had dental problems at the time of the study but chose not to seek dental care. According to the subjects' visitation patterns, roughly 20% had not seen a dentist in more than 2 years, and another 20% had never visited a dentist (9).

Diet and diet-related risks play a significant role in the incidence of obesity and dental caries. These diet factors include dietary habits frequencies, consumption of large amounts of fermentable carbohydrates, junk foods, and highly cariogenic diets (41).

Sugars are the most important and the most studied dietary factor in developing dental caries. In this study, there was a significant positive correlation between both dmft and DMFT with the consumption of desserts (cakes/ doughnuts/ sweetened pies/ baklawa/ basbosa/ konafa). Another significant positive correlation was found between deft and the consumption of dairy ice cream, candies, and crackers. The form (solid or liquid) of the fermentable carbohydrate directly influences the duration of exposure and retention of the food on the teeth. Thereby, prolonged retention of cariogenic components on teeth surfaces causes acid production with subsequent early teeth demineralization and caries initiation and propagation (83).

Previous studies revealed that fluid consumption patterns have changed over the past decade with rising consumption of sugary drinks and less consumption of water and milk (84, 85). In this study, the consumption of sweetened juices was found to be associated with all caries indices: dmft, deft, and DMFT. A recent systematic review (86) declared the need to control, limit, and manage the intake of sweetened juices, as they could degenerate not only an adult's dental health but one's overall wellness. Furthermore, Kim et al. (87) recorded in their study conducted in the United States, that among the studied subjects, 26% of young adults lost one or more teeth in association with massive intake of sweetened juices. Interestingly, frequent and prolonged bottle-feeding practices, as well as high consumption of sweet drinks during weaning, could be associated with the formation of early childhood caries (88). It is important to increase the awareness among parents concerning bottle-feeding practices and weaning diet contents and highlight the potential implication for their children's oral health.

In this study, there was a significant positive correlation between dmft and DMFT with soft drinks. It was reported that children with a high consumption rate of soft drinks have undesirable eating patterns and eat high amounts of sugars from other dietary sources. Such an unhealthy diet pattern affects both the primary and permanent dentition (89–91). In contrast, this disagrees with our previous work (47), which recorded a non-significant correlation between soft drinks and dental caries.

A statistically significant negative correlation was detected between dmft and dairy products (unsweetened milk/ cheese/ yogurt). These findings are in agreement with our previous work (47). Egyptian children who consume milk more frequently possessed lower caries index. Milk has low cariogenic potential as it contains calcium, phosphorus, and casein, which are considered caries protective factors (83, 90).

The inverse significant association between fruit/vegetable consumption and caries in all age groups agrees with a study that recorded that dental caries prevalence was higher in non-vegetarians than vegetarians (92). This association could be explained by a lower tendency to consume sweets between meals in vegetarians than non-vegetarians (93). In addition, the naturally occurring sugar within whole fruits, vegetables, some grains, and dairy products is less likely to be overconsumed and is counteracted by the wide range of bioactive health-enhancing nutrients, antioxidants, fiber, and phytochemicals present in fruits/vegetables that can reduce inflammation and improve endothelial function (94).

It has been authorized that a lower risk for first permanent molars caries was found in children consuming diet rich in red meat, poultry, organs, eggs, and seafood (95). Moreover, there was an inverse significant association between meat/poultry/fish and dmft (p <0.05) in this study. These results are concomitant with a study carried out in Zhejiang Province, exploring the prevalence of dental caries in 6- to 8-year-old children, especially their first permanent molars (95).

Obesity and dental caries are multifactorial in etiology as multiple genetic and environmental factors may impact them. Consequently, these confounding factors, including age, gender, lifestyle, and oral hygiene habits, might control their incidence (96).

Individual body weight relative to population norms was evaluated by calculating BMI using the formula BMI = weight in kilograms/height in meters squared (97). Our study found a positive correlation between increased weight represented through BMI and both dmft and deft. This finding contradicts our previous findings, where no correlation between BMI and all caries indices was reported (47). Overweight–obese children have a higher risk of developing dental caries than normal-weight children (98) as they tend to consume high levels of cariogenic and obesogenic food and drinks (26). These findings are confirmed by our results where a significant correlation was recorded between overweight/obesity and the consumption of soft drinks, fast foods, dairy ice cream, granulated sugars, and desserts (cakes/ doughnuts/ sweetened pies/ baklawa/ basbosa/ konafa).

In this study, 66.9% of the studied population were overweight/obese. This percentage is higher than what has been reported in our previous work in 2020, as the percentage of overweight/obese children in the same age group was 44.4% (99). The small sample size for this age group (5–9 years) in the previous work as well as the change in the dietary habits with the time could explain this discrepancy. In this work, a higher percentage of male participants were overweight/obesity than females, and BMI was significantly positively correlated with soft drinks. These findings support the results of our previous work (99).

A statistically significant positive correlation was detected between BMI and legumes. This result was in disagreement with a cross-sectional study conducted by Papanikolaou et al. (100), which concluded that legume consumption is related to healthier body weights, as legumes are not considered energy-dense food, they are low in dietary fat (101) and rich in dietary fibers (102). Since bread is an important dietary constituent and legumes are usually consumed with bread in the Egyptian diet, this combination might explain the positive correlation between BMI and legumes.

A statistically significant negative correlation was detected between BMI and meat/poultry/fish, fresh fruits, and vegetable consumption. This finding supports previous report that obese children's diet is usually low in fiber (103) and proteins, with protein-deficient malnutrition, while their energy intake comprises high carbohydrates and highly processed foods (104).

The literature reports on the association of dental caries with BMI are conflicting, and three systematic reviews (53, 105, 106) found inconclusive evidence regarding the association between obesity and dental caries. The possible reason (81) for the conflicting reports is that dental caries and BMI are non–linearly related, with more dental caries occurring in individuals with either higher or lower BMI. Thereby, we suggest that methodological factors together with sample demographics, dental examination accuracy, and data analysis methodology affect whether or not the association is detected.

There is a growing evidence that emphasizes the role of physical inactivity in the development of obesity among children and adults (26, 107, 108). This comes in accordance with our results, a statistically significant negative correlation was detected between BMI and healthy lifestyle habits.

There are multiple social and environmental factors linked to the purchase and consumption of sugar-sweetened beverages and junk food. Policies aiming to reduce their consumption should be addressed. First, regulating food advertising and promotion since there is a growing evidence on the relation between food advertising and increased intake of calorie-dense products by adult populations (109) and children (110). Second, labeling of sugar-sweetened beverages and raising awareness about their health effects. A systematic review on the impact of front-of-package labeling on consumption suggested that consumers identify healthier foods more easily from nutrient-specific schemes (111). Third, school interventions and nutrition policies to restrict the availability of sugar-sweetened beverages and provide healthier food in schools (112). Finally, imposing taxes on sugar-sweetened beverages. Higher prices of sugar-sweetened beverages, food taxes, and subsidies may affect their consumption rate and improve health outcomes (113–116). Among EMR, 15 countries have developed either a policy or prepared a draft to reduce the impact of marketing of unhealthy foods and beverages to children in educational facilities and on media. These countries include all high-income countries (GCC), middle-income countries (Egypt, Iran, Jordan, Lebanon, Morocco, Pakistan, and Palestine), and a low-income country (Yemen) (110, 117). Taxes on sugar-sweetened beverages have been implemented in all high-income countries (GCC) and middle-income countries including Egypt, Iran, Morocco, Palestine, and Tunisia. Front-of-pack labeling has been applied in five countries in EMR, Saudi Arabia, UAE, Iran, Morocco, and Tunisia (117).

Taken with this findings, both overweight/obesity and dental caries have mutual determinants and require an inclusive, integrated management approach by multidisciplinary medical teams. BMI calculation should be included in the standard dental evaluation of any pediatric patient, providing a screen for prevention, timely diagnosis, and treatment of the children suffering from dental caries and malnutrition (overweight/obesity). Soft drinks, sweetened juices, and sweetened snacks, which are frequently available at schools, are associated with overweight/obesity and dental caries. Hence, health promotion activities should highlight the importance of reducing the consumption of these unhealthy food items and other sugary foods in homes and schools.

## Conclusion

Investigating the dietary habits of the Egyptian school children revealed a positive correlation between desserts (sweetened snacks), soft drinks, and the incidence of dental caries and overweight/obesity; conversely, the consumption of fruits and vegetables was negatively associated. Sweetened juices were positively associated with the three caries indices. Overweight/obesity was significantly positively correlated with primary dentition caries; accordingly, BMI calculation should be included in the routine examination in dental clinics. Despite the forward step taken by the Egyptian government by applying taxes on soft drinks, more taxes need to be applied on other sweetened beverages, including juices and milk. Moreover, legislation regulating the sale of these food items and prohibiting the marketing of these unhealthy food items in school canteens is mandatorily needed. In addition, the results of this study have identified children as a target group of the population to apply interventional and preventive strategies against dental caries and obesity. The government should educate parents about healthy dietary habits, dental health practices, and the benefits of physical activity through different media approaches. Further studies assessing the level of parents' awareness in more detail are needed.

## Data availability statement

The raw data supporting the conclusions of this article will be made available by the authors, without undue reservation.

## Ethics statement

The studies involving human participants were reviewed and approved by the Research Ethics Committee of Cairo University's Faculty of Dentistry's requirements (Approval: 24920). Written informed consent to participate in this study was provided by the participants' legal guardian/next of kin. Written informed consent was obtained from the individual(s), and minor(s)' legal guardian/next of kin, for the publication of any potentially identifiable images or data included in this article.

## Author contributions

All authors listed have made a substantial, direct, and intellectual contribution to the work and approved it for publication.

## Conflict of interest

The authors declare that the research was conducted in the absence of any commercial or financial relationships that could be construed as a potential conflict of interest.

## Publisher's note

All claims expressed in this article are solely those of the authors and do not necessarily represent those of their affiliated organizations, or those of the publisher, the editors and the reviewers. Any product that may be evaluated in this article, or claim that may be made by its manufacturer, is not guaranteed or endorsed by the publisher.
